# Improved binaural speech reception thresholds through small symmetrical separation of speech and noise

**DOI:** 10.1371/journal.pone.0236469

**Published:** 2020-08-05

**Authors:** Luise Wagner, Lukas Geiling, Christopher Hauth, Thomas Hocke, Stefan Plontke, Torsten Rahne

**Affiliations:** 1 Department of Otorhinolaryngology, Head and Neck Surgery, Martin Luther University Halle-Wittenberg, University Medicine Halle, Halle, Germany; 2 Department of Medical Physics and Cluster of Excellence Hearing4All, Carl von Ossietzky University, Oldenburg, Germany; 3 Cochlear Deutschland GmbH & Co. KG, Hannover, Germany; Medical University Hannover; Cluster of Excellence Hearing4all, GERMANY

## Abstract

Speech perception in noise is challenging and is improved by binaural hearing. Since signal processing of assistive hearing devices often modifies or masks the peripheral binaural head-shadow or better-ear effects, central binaural processing should be measured separately. In a prospective study, 10 listeners with normal hearing were tested with the German matrix sentence test in a set-up with two loudspeakers located at opposite angles in the horizontal plane with respect to S_0_N_0_. The speech reception threshold (SRT) was investigated depending on the separation angle between speech and noise. The lowest (best) SRT was obtained for a separation of target and interfering source from S_0_N_0_ at an angle of about S_±60°_N_∓60°_. The derived normative curve was comparable to SRTs predicted by the binaural-speech-intelligibility-model. The systematic separation of signal and noise showed a significant improvement in speech intelligibility for normal-hearing people even for small separation angles. This experimental setting was verified. This study aimed to assess the effect of small sound source separation on binaural hearing and speech perception.

## Introduction

Understanding speech in noisy environments is challenging even for listeners with normal hearing and becomes even more difficult if the surrounding noise is competing speech [1,2]. Hearing-impaired listeners, including users of assistive hearing devices, are more affected than normal hearing listeners [3–5]. Understanding the basal processes a bit better might help to improve device fitting. Binaural hearing has a huge positive effect on speech perception in spatially distributed noise and is the focus of many trials for users of hearing technologies [6–8]. The binaural neural processing of the interaural level difference (ILD) and interaural time difference (ITD) as, e.g. described by the classic equalization-cancellation (EC) model [9] improve the internal signal-to-noise ratio (SNR) and thus the speech intelligibility [10]. In conditions with spatially separated sound sources the sound shadow of the head results in one ear with a better SNR. This so called better-ear-effect improves speech intelligibility [11]. It is further referred to as the ear with the better speech reception threshold (SRT) in noise [12,13] in the tested situation.

Beside the monaural assessment of the head shadow effect, binaural signal processing may result in binaural summation, which is the difference in SRT between listening with two ears and listening with the better monaural ear, or the binaural squelch [14]. The binaural squelch effect describes the benefit in speech intelligibility that is obtained when adding an ear with a poorer SNR, assuming that both ears have the same monaural performance [8]. The two aspects of the better-ear effect can be considered as rather passive effects *per se*, while the binaural summation and even more the squelch effect might be viewed as higher order influence in the auditory processing pathway [7].

In people with hearing loss using assistive hearing technologies the ability to process ILD and ITD is limited [15]. Binaural summation effect, head shadow effect, and squelch effect are significantly smaller than in listeners with normal hearing [7]. The high variability in assistive technology parameters, e.g., processing times and channel numbers, and the asymmetry of hearing performance between the ears often result in bad outcome because of the wrong input in the auditory system caused by the hearing devices. Also synchronization technologies does not always help. Klicken oder tippen Sie hier, um Text einzugeben.Therefore, measuring binaural effects is challenging [12,13].

For clinical testing, a procedure is needed which reduces the influence of the rather peripheral effects and focus on the central binaural effects. Typical sound source settings for the evaluation of binaural hearing with speech in noise tests range from a simple S_0_N_0_ presentation for binaural summation to multiple loudspeaker settings [16] and multisource noise fields and virtual acoustics [6,17,18]. Other common settings present speech and noise signals from opposite sides or with separation angles of 90° (S_0_N_±90_, N_0_S_±90_) [19].

Gabriel et al. [20] found that even small angles of signal and source separation improve binaural hearing in a dichotic speech perception test. In their study, they focused on binaural processing in test persons with auditory processing disorder under the assumption of symmetrical hearing thresholds. The large differences in hearing performance between the measured age groups indicated that spatial source separation exhibits good diagnostic potential.

In our study on normal-hearing subjects, the head shadow and better-ear effect were minimized by focusing on small symmetrical spatial separation between speech and noise sources, both separated in the frontal position. The SRT improvement as a function of separation angle determines the benefit of spatial separation of signal and noise and might serve as a tool for evaluating hearing aid and speech processor settings which are presumed to provide some improvement to the user [21].

The head shadow effect was assessed separately by measuring the speech and noise separately in a binaural dummy head recording and using a binaural speech intelligibility model (BSIM). To our knowledge, BSIM was used for the first time to predict SRT for symmetrically separated sound sources [22].

This study aimed to assess the effect of small sound source separation on binaural hearing and speech intelligibility. A normative curve of SRT improvement for normal-hearing subjects was recorded as well as the prediction of BSIM for the measured setup verified.

## Material and methods

### Participants

We conducted a prospective experimental trial involving ten healthy adults with normal hearing (age 21 to 26 years, five males, five females). Their pure-tone hearing level threshold averaged across 0.5–4kHz was between 1.25 and 11.25 dB (mean [M] = 6.5 dB; SD = 2.7 dB) with a maximal side difference of 6.25 dB. The maximal threshold was 20 dB for one participant at 6 kHz, any other thresholds did not exceed 15 dB. Participants had no acute or chronic ear diseases or health conditions relevant to the study question (e.g., no middle ear disease, history of extensive noise exposure in the past or treatment with ototoxic drugs, central nervous system or cardiovascular disorders and no known metabolic diseases such as diabetes, hyperlipidemia or autoimmune diseases). All participants signed a written informed consent. The protocol for this study was according to the Declaration of Helsinki. It was approved by the Ethics Committee of the Martin-Luther-University Halle-Wittenberg (ethics committee approval number: 2019–028).

### Stimuli

We used the German matrix sentence test (Oldenburg sentence test, OLSA [23]) to measure the SRT in noise. The SRT was defined as the signal-to-noise ratio (SNR) when 50% of the presented items are understood correctly. We used the “OLnoise” which is a 30 times random-phase superposition of the whole speech material [23]. Noise and speech were presented with a sound pressure level of 60 dB at the beginning of each test list. The speech level was adapted according to the described procedure in equation 9 of Brand and Kollmeier [24] while the noise level was kept constant.

### Procedure and sound sources

**Fig 1:**
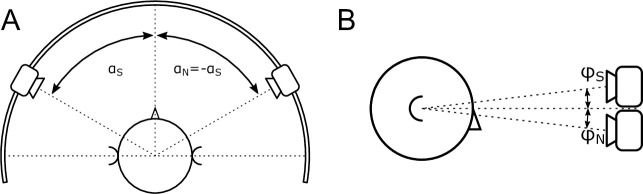
Setup configuration with the symmetrical azimuth position (A). Negative values for “a” indicate a signal from left. Up to an azimuth of 17°, the sound sources were also vertically separated because of the geometrical size of the speakers (*φ*) (B). S stands for signal and N for noise.

The measurements were done in a sound insulated room with a reverberation time of 0.35 s. All sound stimuli were generated by a PC, converted by Fireface 400 interface (RME Audio AG, Haimhausen, Germany) to an analog signal and sent to a POA-800 power amplifier (Denon KK, Kawasaki, Japan). Two CD 1020 loudspeakers (Canton, Weilrod, Germany) were mounted in a custom-made semicircle in the horizontal plane (Fig 1). The angle could be adjusted continuously in a range from 0° to ±95° with an accuracy of 0.5°. The participants were seated in a chair (Modell CL 11/1, Der Drehstuhl, Berlin, Germany) in a fixed position at a distance of 1 m from the speakers. The height of the loudspeakers was adjusted to each participant’s external ear canal level by use of a positioning laser. The participant’s head position was fixed to a fixation construction (Papillon, Focal Meditech BV, Tilburg, Netherlands).

To familiarize the participants with the test and reduce the training effect, two sentence lists with 20 sentences each were presented prior to the actual measurement. This step was followed by tests with systematic, symmetric separations of the loudspeakers with angles of 0°, 1°, 2°, 4°, 6°, 9°, 13°, 20°, 30°, 45°, and 90°. Additional presentations at 60° were applied for five random participants and at 75° for three random participants, respectively. The order of presentation was inverted for five of the ten participants.

Because of the geometrical dimensions, it was not possible to adjust the loudspeakers at the same height in the frontal position (0°; Fig 1B; *φ* = ±2.8°). Therefore, the influence of the height adjustment for angles larger than 6° was investigated. It was changed for five participants at an angle of ± 9°, and for the other five, it was kept constant for the entire measurement for all angles. For six participants, speech was presented on the left and the noise on the right side, and for four participants, this was reversed.

### Data analysis

The change in speech reception threshold by sound source separation in reference to the SRT at S_0_N_0_ (ΔSRT = SRT(S_±x_N_∓x_) –SRT(S_0_N_0_)) was calculated for each loudspeaker position and averaged over all participants. Since the noise and speech directions were randomized between the subjects SRTs were compared between the sides by an analysis of variances (ANOVA). The averaged SRT over both sides were then compared between the presentation angles by an ANOVA for repeated measures. Bonferroni corrections were used for post hoc comparison.

The angle at which the SRT improvement reached significance and the angle with the lowest (best) SRT were identified. Since a SD for the test–retest differences in e.g., cochlear implant users of around 1 dB was reported [25], we assumed an improvement of 2 dB as being relevant. The angle at which an SRT improvement of 2 dB was reached was calculated. Since the focus was laid on small angles, the slope of the SRT improvement was fitted with a linear function up to an angle of ±13°. SPSS software version 25 (IBM, Germany) was used for all statistical analysis.

### Binaural speech intelligibility model

For comparison of the observed results with model predictions, the binaural speech intelligibility model (BSIM) was used [22]. For this study, the speech and noise signals were recorded separately for all measured angle positions using a KU 100 head simulator (Neumann, Berlin, Germany). BSIM filters the input signals using a gammatone filter bank [26] ranging from 146 Hz to 8300 Hz, simulating the frequency selectivity on the basilar membrane. After peripheral filtering, binaural processing is considered using the equalization–cancellation (EC) mechanism [9]. In the EC mechanism, the ITDs and ILDs are equalized between the left and right ear channels before the cancellation step (subtraction) is applied. The equalization parameters are chosen in a way, that the SNR at the output of the EC mechanism is maximized. For the binaurally processed signal, the SNR is calculated and compared to the SNR at the left ear and right ear. The maximum SNR is considered in the speech intelligibility index [27], where the frequency-dependent SNRs are weighted according to human speech perception and transformed to an index value between 0 and 1. To predict the SRT for different azimuths of speech and noise, for this experiment a reference SII value of 0.23 was determined, which usually corresponds to the SRT obtained for speech and noise coming from 0° azimuth. This is comparable to other SII values of 0.2 [22], 0.201 [28] and 0.24 [29].

## Results

**Fig 2:**
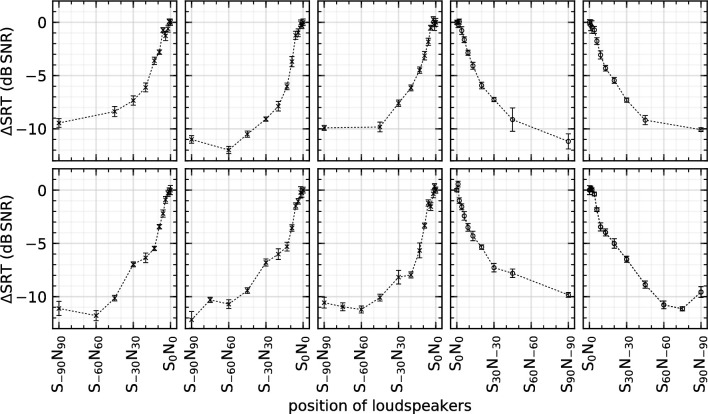
Individual ΔSRT (speech reception threshold) for all 10 participants at different loudspeaker positions (i.e., separation of sound sources). The speech signal is presented from the left (crosses) or from the right (circles) and the noise with the same azimuth but from the opposite sides. Error bars show the fitting error of the adaptive SRT measurement.

At S_0_N_0_, the mean SRT across all participants was –7.3 dB (SD = 2.2 dB) SNR relatively to a noise level of 60 dB SPL. Fig 2 shows the ΔSRT for each participant. The different elevations of the loudspeaker show no significant difference in the resulting SRT for angles larger than 6°. The worst SRT was always at S_0_N_0_ and used as reference (ΔSRT = 0). For all participants, the trend of the curve was the same, and the ΔSRT improved when the sources were separated. At approximately S_±60°_N_∓60°_, the SRT reached a plateau or a minimum and in most cases increased slightly until the maximum separation angle of S_±90°_N_∓90°_ in this experimental series.

**Fig 3:**
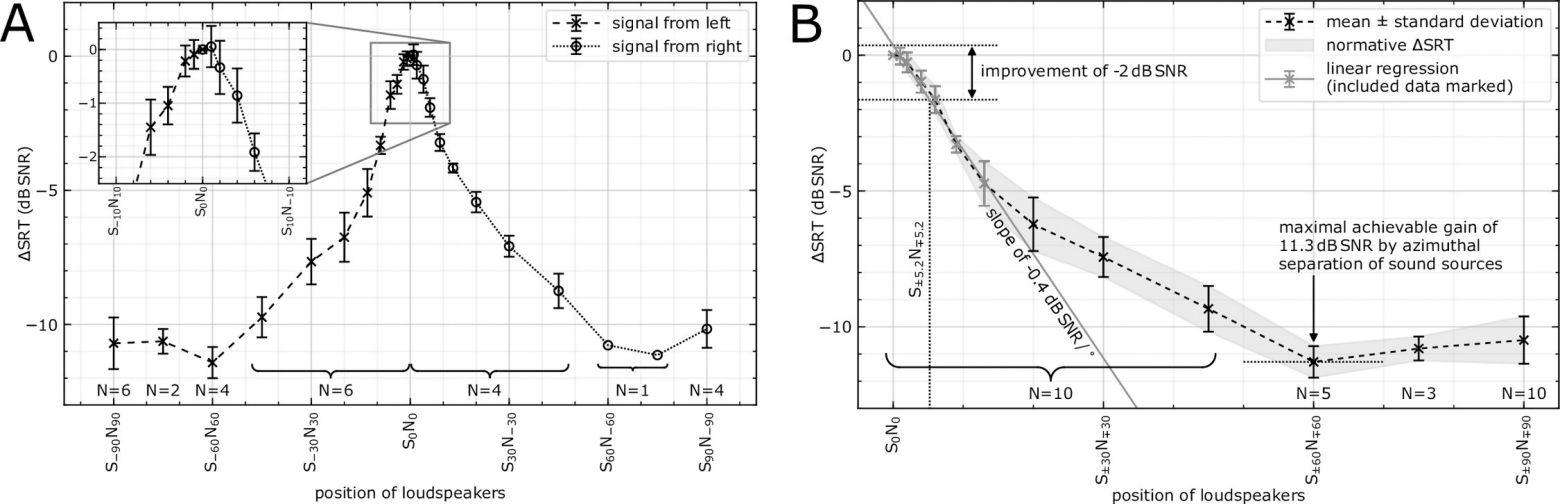
Averaged ΔSRT (speech reception threshold) if speech is presented from the left and right sides (A) and mirrored and averaged for all 10 participants (B). An improvement of –2 dB SNR was found for a separation of 10.4° and a maximal improvement of –11.3 dB SNR for angles about 60°, i.e., a separation of 120°. For angles up to 13° (a separation of 26°), an improvement of –0.4 dB SNR/° was observed. The grey area between the ΔSRT and its standard deviation is in the following referred to as normative area.

The averaged ΔSRT values are shown in Fig 3. Because there was no significant difference between the sides (Fig 3A), results of the left side were inverted and averaged (Fig 3B). An ANOVA showed a significant effect of angle (F(10, 90) = 469.8, *p*<0.001). Post hoc comparisons showed that increased angles led to reduced SRTs. SRT measures with angles from 4° and above were significantly different from the S_0_N_0_ condition (all *p*s<0.01).

The figure also shows the SNR improvement of 2 dB at a loudspeaker position of S_±5.2°_N_∓5.2°_. Thus, a symmetric separation of signal and noise in the front of 10.4° led to an SRT improvement of 2 dB SNR. For small angles up to 13° (i.e., a symmetric separation of signal and noise of 26°), the improvement can be described with a linear fit with a slope of –0.4 dB SNR/°. Fig 3B also shows that the maximal improvement in speech intelligibility compared to S_0_N_0_ was 11.3 dB SNR (SD: 0.58 dB SNR), which was measured at the S_±60°_N_∓60°_ loudspeaker position (120° symmetric separation of signal and noise).

**Fig 4:**
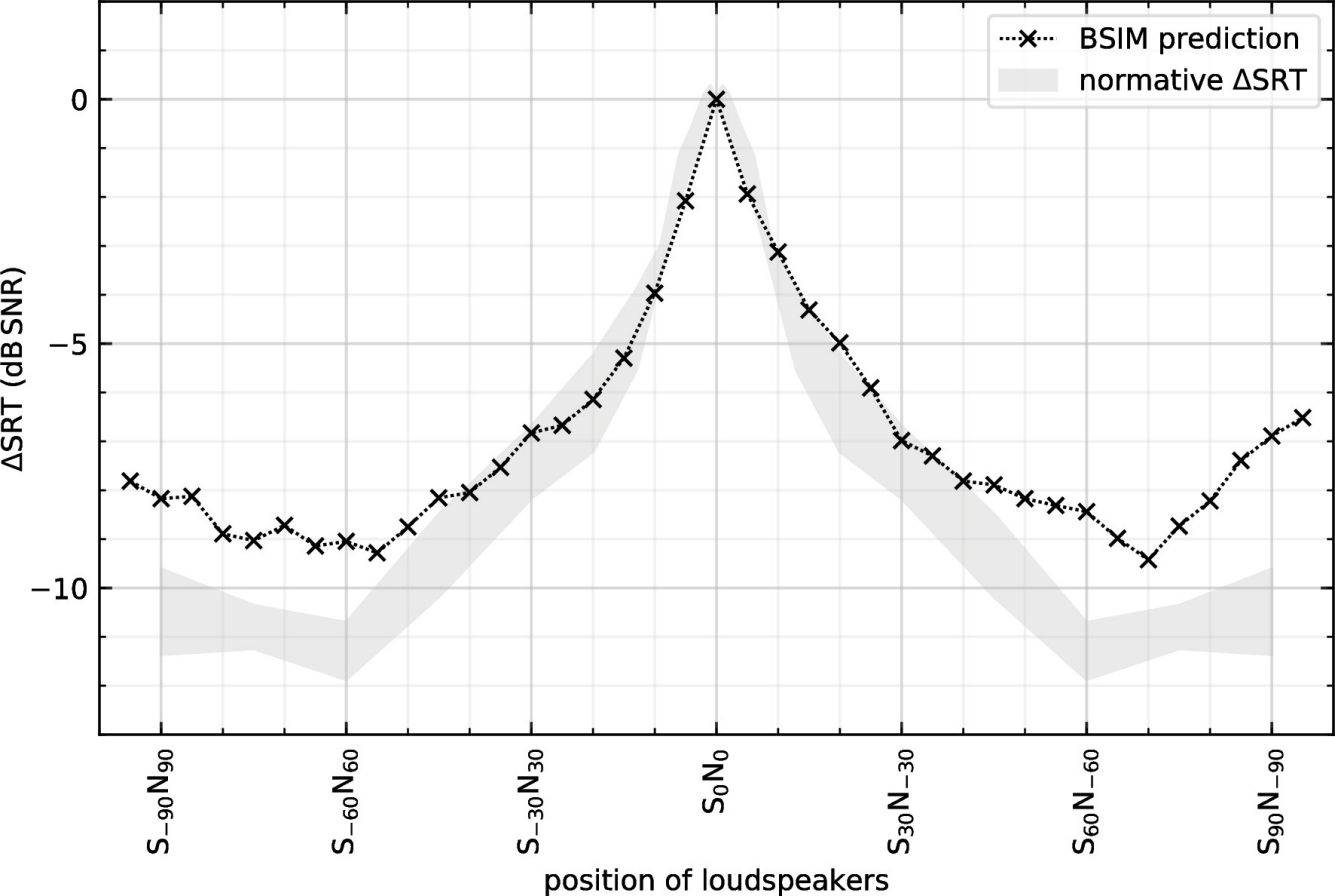
The comparison for the predicted Binaural Speech Intelligibility Model (BSIM) with the results of the averaged normative area shows a good prediction up to a sound source separation of about 80°.

Measured binaural dummy head recording was used to calculate the BSIM. Fig 4 shows the SRT predicted by the BSIM as well as the normative curve derived from Fig 3. The area between the ΔSRT and its standard deviation of Fig 3B is referred to as the normative curve in the following. It has to be addressed that these results are derived from a measure in a not completely anechoic chamber and for use as normative data the reverberation time has to be considered. Further these “normative” data are only valid for continuous maskers with a frequency spectrum of the target.

Up to an azimuth of 40°, the model data is within the range of measured normative data.

## Discussion

This study aimed to assess the effect of small sound source separation on binaural hearing and speech perception.

Under the frontal S_0_N_0_ presentation of both, signal and noise, the SRT was –7.3 dB SNR, which is similar to values of –7.1 dB SNR reported for the used speech test by others [23]. The results demonstrate a significant SRT improvement if speech and noise sources were separated symmetrically. The measured maximum SRT improvement with the continuous masker with frequency spectrum of the target was 11.3 dB SNR and reached at a symmetrical source separation of 120° (angle of ±60° for signal and noise) with a plateau at larger angles. This result was expected since ILDs increase monotonically up to angles of 60° [30] and also binaural unmasking does not increase beyond 60° [31]. This improvement can be described with geometrical properties like the position of the ears relative to the head or the shape of the ear and pinna, which optimize sound localization for the frontal part of the horizontal plane [32]. With this measure we found the maximal improvement possible for normal hearing people. This therefore identifies the maximal target SRT improvement that could and should be reached with assistive hearing devices.

We are aware of the limitations we are faced with because of the small group of participants, fixed speaker positions and a non-random presentation of the stimuli. To get more precise results the number of participants can be increased. Smaller steps in changing the angles would lead to a better resolved curve. Nevertheless the data already fit to the predicted BSIM data. Although the SRT improvement was not unexpected, it could be shown, for the first time, for symmetrical separation of sound sources with high angle resolution. The SRT predicted by the BSIM fit to the measured SRT up to sound source separations of ±40° (Fig 4). Thus, our study verifies the predictability of SRT thresholds by the BSIM also for arbitrary symmetric angle separations. For higher angles there is a slight deviation between prediction and measured data. For angles between 5° and 45° the gain by EC is largest for higher angles it decreases. The masking effect is for larger angles smaller. An effect of the head simulator might be also seen here.

The high angle resolution was used to focus on small symmetric angle separations where the head-shadow is very small. Even for small angles, the SRT improved linearly with the separation angle. The improvement was –0.4 dB SNR / degree. A significant improvement compared to the frontal presentation of both sound sources was reached at an angle of 4°. Those improvements are often within the standard deviations for the test–retest differences in e.g., cochlear implant users of around 1 dB [25]. However since this 1 dB was derived for monaural S_0_N_0_ presentation, we assume an improvement of 2 dB as being relevant. This improvement was already achieved at a relative small source separation angle of 10.4° (±5.2°). Nevertheless this size of 2 dB improvement has to be further investigated for future setups with hearing impaired patients.

The results and experiences from the experimental procedure could be translated to patients with hearing aids, cochlear implants or binaural listeners to assess binaural SRT improvements. It could be the basis for evaluation of beamforming algorithms or future developed algorithms. By including small symmetrical source separations more sensitive information about binaural benefits would be recordable than with the commonly used sound presentations at S_0_N_0_ or Intelligibility Level Difference tests. Even if only a few angles would be used for sound presentation the long recording time would limit such application in clinical routine and might be decreased by using the Oldenburg sentenced test for children (OLKISA) matrix test, which reduces the presented items to 60% as compared to the OLSA used in this study. Since the use of small presentation angles requires a stable head orientation in the room, a good fixation of the head position in the sagittal plane (0°) as done in this study appears crucial.

In their recent cohort study, Hoppe et al. [13] showed that hearing performance in aided hearing-impaired listeners can typically be considered as asymmetrical. Against this background [33,34], a quantification of binaural hearing is needed for evaluation of the treatment. Numerous approaches have attempted to assess hearing performance in asymmetrical hearing conditions. However, the experimental setup is often adapted and therefore limited for the study of particular technical features of hearing aids and/or implants, e.g. adaptive beamforming algorithms that provide the largest improvements in classical S_0_N_90_ settings.

The strength of the proposed setting is the assessment of a binaural SRT as soon as it appears at small angles without being superimposed by the above-mentioned larger “passive” head shadow or better-ear effects. The experimental setting has the potential to support evaluation of fitting procedures and other methods with a focus on binaural hearing. The standardized S_0_N_0_ or S_0_N_90_ setups show constraints in investigating those effects. Future studies should investigate its feasibility in patients with hearing loss. Following that, the development and evaluation of fitting procedures and other methods with a focus on binaural hearing might be supported.

## Supporting information

S1 Rawdata (PDF)

S2 Rawdata (PDF)
