# Correction: Improved binaural speech reception thresholds through small symmetrical separation of speech and noise

**DOI:** 10.1371/journal.pone.0251766

**Published:** 2021-05-11

**Authors:** The PLOS ONE Staff

There is an error in the third paragraph of the Introduction. The correct paragraph is: In people with hearing loss using assistive hearing technologies the ability to process ILD and ITD is limited [15]. Binaural summation effect, head shadow effect, and squelch effect are significantly smaller than in listeners with normal hearing [7]. The high variability in assistive technology parameters, e.g., processing times and channel numbers, and the asymmetry of hearing performance between the ears often result in bad outcome because of the wrong input in the auditory system caused by the hearing devices. Also synchronization technologies does not always help. Therefore, measuring binaural effects is challenging [12,13]. The publisher apologizes for the error.
