# Supplementary material for: Improved binaural speech reception thresholds through small symmetrical separation of speech and noise
Source: PLoS One. 2020 Aug 5;15(8):e0236469. doi: 10.1371/journal.pone.0236469 (PMC7406049; doi:10.1371/journal.pone.0236469)
Supplement: S1 Rawdata — (PDF) [file pone.0236469.s001.pdf]

Raw data of ΔSRT measurements for figure 2 and data basement for figures 3 – 5. The subject number corresponds to the plots shown in figure 2 from left to right and top to bottom respectively. For some subjects the measurements at certain loudspeaker positions were conducted two times. These values are mean values and are marked with an asterisk (*) in the table. For the first 5 subjects the elevation was adapted to equal height for higher separation angles. For the others subjects (6 – 10) the elevation of loudspeakers was kept constant.

subject number	1	2	3	4	5	6	7	8	9	10
sex	male	male	female	female	male	female	female	male	female	male
age	21	24	26	25	22	23	22	24	22	23
side	left	left	left	right	right	left	left	left	right	right
adaption of elevation	changed	changed	changed	changed	changed	unchanged	unchanged	unchanged	unchanged	unchanged
position of loudspeakers	ΔSRT (dB SNR)									
S-90N90	-9.45(41)	-11.00(35)	-9.91(23)			-11.11(67)*	-12.18(78)	-10.57(51)*		
S-75N75							-10.30(24)	-10.95(37)		
S-60N60		-11.99(34)				-11.78(47)	-10.70(41)	-11.21(33)		
S-45N45	-8.37(47)	-10.50(29)	-9.83(46)			-10.14(26)	-9.44(26)	-10.09(33)		
S-30N30	-7.34(43)*	-9.08(20)	-7.59(30)			-6.97(23)	-6.79(34)	-8.17(64)		
S-20N20	-6.11(42)	-7.88(45)	-6.16(28)			-6.36(45)	-6.01(50)	-7.96(28)		
S-13N13	-3.61(34)	-6.02(31)	-4.49(30)			-5.47(20)	-5.31(41)	-5.66(70)		
S-9N9	-2.80(21)	-3.69(48)	-3.14(39)			-3.43(21)	-3.56(30)	-3.33(24)		
S-6N6	-0.74(23)	-1.22(39)	-1.83(34)			-2.20(34)	-1.49(29)	-1.21(29)		
S-4N4	-1.28(44)	-0.98(36)	-0.51(23)			-0.92(32)	-1.05(27)	-1.55(36)		
S-2N2	-0.60(32)	-0.08(30)	0.26(27)			-0.30(29)	-0.21(53)	-0.35(37)		
S-1N1	0.08(24)	-0.35(24)	-0.28(48)			-0.08(28)	-0.27(39)	0.36(24)		
S0N0	0.00(29)	0.00(31)	0.00(37)*	0.00(20)	0.00(25)	0.00(42)	0.00(28)	0.00(28)	0.00(15)	0.00(42)
S1N-1				-0.21(29)	-0.27(51)				0.56(27)	0.14(23)
S2N-2				0.06(24)	-0.45(59)				-0.99(26)	0.04(18)
S4N-4				-0.77(36)	-0.72(34)				-1.57(28)	-0.37(18)
S6N-6				-1.63(26)	-1.76(31)				-2.42(40)*	-1.84(18)
S9N-9				-2.85(24)	-3.06(38)				-3.50(36)	-3.45(38)
S13N-13				-4.08(33)	-4.31(26)				-4.31(44)	-3.97(33)
S20N-20				-5.93(31)	-5.45(27)				-5.35(21)	-5.02(45)
S30N-30				-7.25(19)	-7.31(20)				-7.28(39)	-6.49(28)
S45N-45				-9.13(110)	-9.18(43)*				-7.81(40)	-8.87(34)*
S60N-60										-10.77(36)*
S75N-75										-11.14(19)
S90N-90				-11.18(71)	-10.08(18)*				-9.83(23)	-9.57(50)*
