# Supplementary material for: Improved binaural speech reception thresholds through small symmetrical separation of speech and noise
Source: PLoS One. 2020 Aug 5;15(8):e0236469. doi: 10.1371/journal.pone.0236469 (PMC7406049; doi:10.1371/journal.pone.0236469)
Supplement: S2 Rawdata — (PDF) [file pone.0236469.s002.pdf]

Raw data for figures 4 and 5. Values marked with an asterisk (*) represent mean values.

| subject number | | 11 | – |
|---|---|---|---|
| sex | | male | – |
| age | | 24 | – |
| side | | both | – |
| adaption of elevation | | unchanged | – |
| method | OLSA | OLKISA | BSIM prediction |
| position of loudspeakers | ΔSRT (dB SNR) | | |
| S-95N95 | | -10.38(78) | -7,81 |
| S-90N90 | -8.96(36) | -9.68(78) | -8,17 |
| S-85N85 | | -10.78(52) | -8,12 |
| S-80N80 | -11.83(24) | -12.62(63) | -8,89 |
| S-75N75 | | -13.22(27) | -9,03 |
| S-70N70 | -12.47(52) | -14.32(70) | -8,72 |
| S-65N65 | | -12.00(52) | -9,14 |
| S-60N60 | -11.72(37) | -10.67(44) | -9,05 |
| S-55N55 | | -10.48(90) | -9,28 |
| S-50N50 | -10.50(33) | -10.48(61) | -8,75 |
| S-45N45 | | -11.82(13) | -8,16 |
| S-40N40 | -8.47(26) | -9.35(60) | -8,05 |
| S-35N35 | | -10.32(71) | -7,53 |
| S-30N30 | -7.17(53) | -8.86(98) | -6,83 |
| S-25N25 | | -5.53(63) | -6,67 |
| S-20N20 | -5.48(48) | -6.01(24) | -6,14 |
| S-15N15 | | -4.96(56) | -5,30 |
| S-10N10 | -3.17(34) | -3.26(33) | -3,97 |
| S-5N5 | | -2.37(54) | -2,08 |
| S0N0 | 0.00(26)* | 0.00(46)* | 0,00 |
| S5N-5 | | -1.54(97) | -1,94 |
| S10N-10 | -3.50(25) | -3.94(74) | -3,12 |
| S15N-15 | | -5.71(39) | -4,31 |
| S20N-20 | -7.19(33) | -8.41(73) | -4,98 |
| S25N-25 | | -7.03(23) | -5,91 |
| S30N-30 | -7.73(52) | -8.37(29) | -6,98 |
| S35N-35 | | -9.77(42) | -7,30 |
| S40N-40 | -10.21(61) | -10.82(91) | -7,81 |
| S45N-45 | | -13.13(38) | -7,89 |
| S50N-50 | -10.81(40) | -11.25(47) | -8,17 |
| S55N-55 | | -13.65(35) | -8,31 |
| S60N-60 | -11.26(58) | -14.30(50) | -8,44 |
| S65N-65 | | -11.48(19) | -8,98 |
| S70N-70 | -11.25(67) | -13.73(14) | -9,42 |
| S75N-75 | | -12.76(49) | -8,73 |
| S80N-80 | -10.04(35) | -10.12(45) | -8,22 |
| S85N-85 | | -10.94(50) | -7,39 |
| S90N-90 | -9.30(79) | -14.37(12) | -6,89 |
| S95N-95 | | -12.71(49) | -6,52 |
